# Supplementary material for: A surrogate virus neutralization test to quantify antibody-mediated inhibition of SARS-CoV-2 in finger stick dried blood spot samples
Source: Sci Rep. 2021 Jul 28;11:15321. doi: 10.1038/s41598-021-94653-z (PMC8319431; doi:10.1038/s41598-021-94653-z)
Supplement: Supplementary file 1 — Supplementary Information. [file 41598_2021_94653_MOESM1_ESM.pdf]

SUPPLEMENTARY INFORMATION

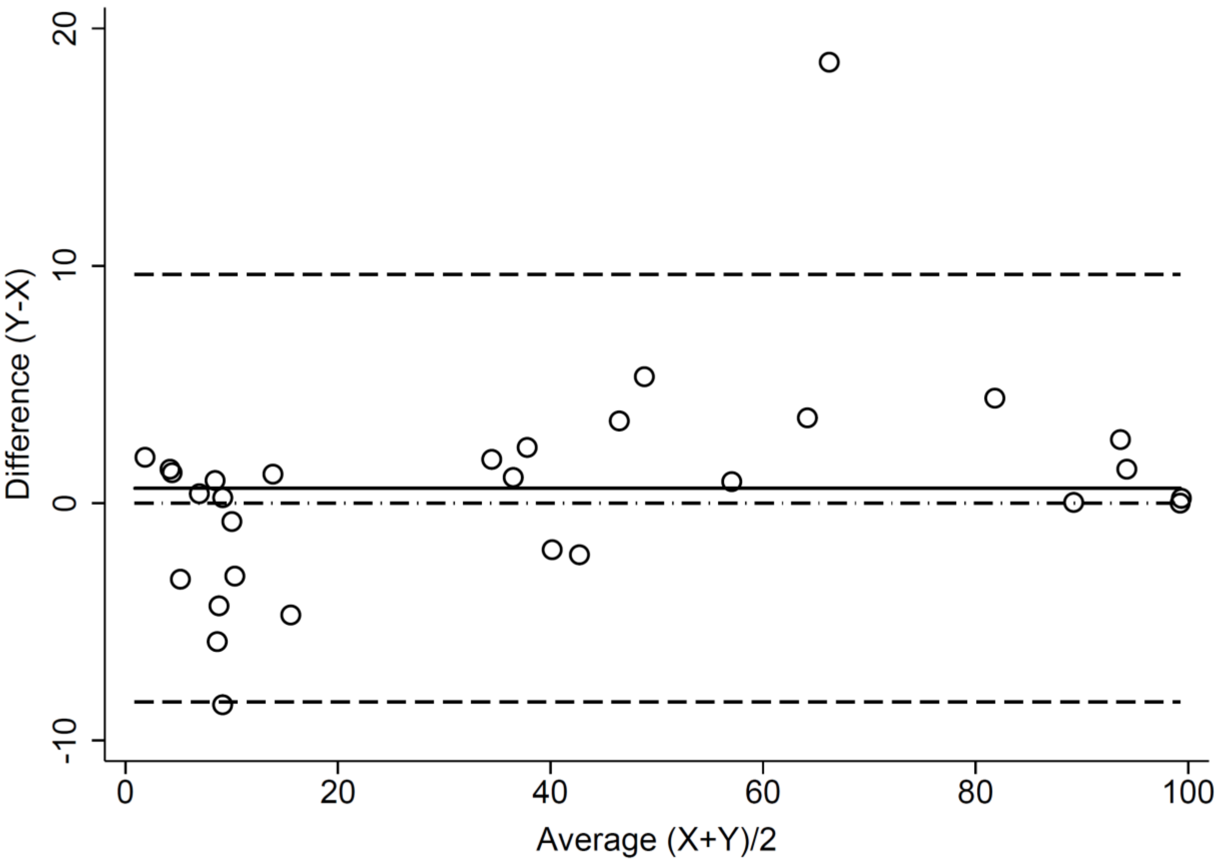

Figure S1. Agreement in neutralization results for matched serum and DBS samples. Bland-Altman difference plot with 95% limits of agreement for results from serum and DBS samples for % neutralization of SARS-CoV-2 spike-ACE2 interaction (X=DBS; Y=serum). Mean bias = 0.63 (95% CI: -1.09, 2.34); lower limit of agreement = -8.38 (95% CI: -11.34, -5.41); upper limit of agreement = 9.64 (95% CI: 6.66, 12.61); concordance correlation of absolute agreement = 0.991.
